# Comparison of vaccine-induced immune thrombocytopenia and thrombosis cases following two adenovirus-vectored COVID-19 vaccines

**DOI:** 10.1038/s43856-025-00891-x

**Published:** 2025-05-10

**Authors:** Rian Van Rampelbergh, Sue Pavord, Luis Anaya-Velarde, Vitalija van Paassen, Karin Hardt, Emiliano Tatar, Javier Ruiz-Guiñazú, Dawn Baumgardner, Valérie Oriol Mathieu, Nicolas Praet, Hendy Kristyanto, Jerald Sadoff, Macaya Douoguih, Yimei Xu, Frank Struyf

**Affiliations:** 1 Janssen Research and Development, Turnhoutseweg 30, Beerse, Belgium; 2 Department of Haematology, Oxford University Hospitals NHS Foundation Trust, St Edmund Hall, Queen’s Lane, Oxford, UK; 3 Janssen Vaccines and Prevention B.V.a, Archimedesweg 29, Leiden2333 CM, Horsham, The Netherlands; 4 Janssen Research and Development LLC, Spring House, 850 Ridgeview Drive, Horsham, PA USA

**Keywords:** Diseases, Haematological diseases

## Abstract

**Background:**

Vaccine-induced immune thrombocytopenia and thrombosis (VITT) was first described after administration of adenovirus-vectored COVID-19 vaccines including Ad26.COV2.S and ChAdOx1 nCoV-19. It is not known if the clinical characteristics and outcomes of VITT after Ad26.COV2.S and ChAdOx1 nCoV-19 vaccination are different. We assessed demographic and clinical characteristics, laboratory findings and outcomes in patients with VITT after each vaccine.

**Methods:**

Spontaneous postmarketing reports of VITT after Ad26.COV2.S were identified from Janssen’s Global Safety Database and classified using NICE criteria (*n* = 86). Cases after ChAdOx1 nCoV-19 were identified from a published case series (*n* = 220). The analysis is descriptive.

**Results:**

The median age of patients with definite/probable VITT after Ad26.COV2.S or ChAdOx1 nCoV-19 vaccination is 43 and 48 years, respectively. Median time-to-onset is 11 days and 14 days post-vaccination, cerebral venous thrombosis (CVT) is present in 50.6% and 50%, and mortality is 30% and 22% of patients, respectively. Women make up 55.3% of cases after Ad26.COV2.S and 55% after ChAdOx1 nCoV-19, 74%/60% of CVT cases and 68%/62.5% of deaths. Patients present with severe thrombocytopenia, grossly elevated D-dimer, and most test positive for anti-platelet factor-4 antibodies. Patients with preexisting rare autoimmune diseases are observed despite the small sample sizes.

**Conclusion:**

Within the limitations of the data, our study finds no strong evidence for a clinically relevant difference in VITT occurring after Ad26.COV2.S or ChAdOx1 nCoV-19. Observed differences in some parameters likely result from the demographic of the populations vaccinated, and the situational and reporting differences in how, when, and where patients were identified and treated.

## Plain language summary

VITT (also called Vaccine-induced immune thrombocytopenia and thrombosis) is a rare side-effect that can follow vaccination with certain COVID-19 vaccines (Ad26 and ChAdOx1) and typically presents with blood clots (also called thrombosis) and low levels of platelets (tiny cell fragments that help blood to clot, referred to as thrombocytopenia when low). We investigated the characteristics of patients who developed VITT after vaccination and found no evidence that VITT after Ad26 was different to VITT after ChAdOx1. Both of these vaccines were administered to millions of people and saved many lives during the COVID-19 pandemic. Vaccination continues to be the most effective way to reduce or prevent severe or fatal infections.

## Abstract (web summary)

Van Rampelbergh et al investigate the clinical characteristics and outcomes of vaccine-induced immune thrombocytopenia and thrombosis (VITT) following vaccination with 2 adenovirus vector COVID-19 vaccines: Ad26.COV2.S and ChAdOx1 nCoV-19. No strong evidence for a clinically relevant difference in VITT occurring after either vaccine.

## Introduction

Vaccine-induced immune thrombocytopenia and thrombosis (VITT) is a new clinical entity first described after the administration of adenovirus-vectored COVID-19 vaccines including Ad26.COV2.S (Janssen, referred to hereafter as Ad26) and ChAdOx1 nCoV-19 (AstraZeneca, referred to hereafter as ChAdOx1), VITT is characterised by the onset of symptoms 5–30 days after COVID-19 vaccination, with thrombocytopenia, thrombosis, presence of anti-platelet factor 4 (PF4) antibodies, and grossly elevated *D*-dimer^1^. Thromboses frequently occur in multiple vascular beds and unusual anatomical sites such as the cerebral venous sinuses, internal jugular and splanchnic veins. Case identification is challenged by the rarity of the disease, lack of awareness, diverse clinical presentations due to varied loci of thromboses, variable access to laboratory investigations, and confusion with thrombotic thrombocytopenic syndromes identified after vaccination that may or may not be causally related^2–6^. As a result, the true incidence of VITT is difficult to establish. Incidence rates of VITT appear to be higher after ChAdOx1 than Ad26^7,8^, a phenomenon potentially contributed to by differences in process-related impurities, with higher levels of anti-PF4 complexes and increased vascular permeability observed after ChAdOx than Ad26^9^. However, accurate comparison of incidence is not possible due to differences in the way each vaccine was deployed in terms of geographical distribution, age and risk groups targeted for vaccination, and disease awareness. Incidence rates have been reported to be highest in women aged 30–49 years^1,7,10^, but sex-specific exposure data for each vaccine to guide interpretation of this observation are limited.

The differential diagnosis of VITT includes other immune-mediated causes of thrombosis with co-occurring thrombocytopenia, such as heparin-induced thrombocytopenia (HIT), antiphospholipid syndrome, and haemolytic-uraemic syndrome, as well as non-immune mediated causes, such as malignancies, liver disease, septicaemia, haemolysis-elevated liver enzymes and low platelets syndrome and drug toxicity. Co-occurring thrombosis and thrombocytopenia may also be associated with infections, such as dengue or cerebral malaria^11^.

There is increasing understanding of the underlying pathophysiological processes of VITT, the nature of the VITT anti-PF4 antibodies and the immune-complex activation of platelets and leucocytes^12–14^. Large scale epidemiological studies have not shown increased thrombosis with mRNA vaccines and VITT has not been reported after vaccination with other Ad26-vector vaccines that have been administered to more than 290,000 recipients^15–17^. These included Ad26-vectored respiratory syncytial virus, Ebola, human immunodeficiency virus, human papillomavirus, and Zika virus vaccines. ChAdOx1 and Ad26 are both replication-incompetent adenoviruses that encode the SARS-CoV-2 spike glycoprotein in the trimeric prefusion confirmation^10,18^. ChAdOx1 is a chimpanzee adenovirus vector produced in genetically modified human embryonic kidney 293 cells, whereas Ad26 is a human adenovirus 26 vector produced using the PER.C6 TetR Cell Line. It is not known if the clinical characteristics and outcomes of VITT after Ad26 differ from those after ChAdOx1. To address this question, we assessed the demographic and clinical characteristics, laboratory findings, and outcomes of VITT occurring after Ad26 and ChAdOx1 vaccinations. We found that neither the characteristics of patients with VITT, nor their outcomes, differed in any clinically meaningful way after ChAdOx1 or Ad26 vaccination.

## Methods

### Identification of VITT cases after Ad26.COV2.S

Janssen’s Global Safety Database receives spontaneous adverse event reports from worldwide sources including patients, healthcare professionals, pharmacists, lay persons, clinical trials and regulatory agencies. Spontaneous reports of co-occurring thrombosis and thrombocytopenia were identified from the database (data cutoff date: October 10 2022) using standardised Medical Dictionary for Regulatory Activities (MedDRA) (MedDRA, version 24.1) queries (SMQs). All cases with preferred terms that fell within the following search terms were identified: embolic and thrombotic events (SMQ); haematopoietic thrombocytopenia (SMQ, broad), or thrombocytopenia (high-level term), using the same search terms. To ensure that all events of thrombocytopenia were identified, a manual word search was conducted within the case narratives of one of the queries (Query A: Embolic and thrombotic events SMQ) for synonyms or concepts related to thrombocytopenia. Cases qualified for further assessment if thrombocytopenia was reported in temporal association (within a 42-day window) with the thrombotic event. Data were transferred to SAS studio Release: 3.8 (Enterprise Edition), SAS release: 9.04.01M6P11072018.

To allow direct comparison with a previously published case series of VITT after ChAdOx1 vaccination from the United Kingdom^1^, all cases were classified using criteria published by the National Institute for Health and Care Excellence (NICE) (Table 1)^4^. Definite VITT was defined as a case meeting all five of the following criteria: 1) onset of symptoms 5–30 days after vaccination against SARS-CoV-2 (or ≤42 days in patients with isolated deep vein thrombosis or pulmonary embolism); 2) presence of thrombosis; 3) thrombocytopenia (platelet count <150 × 10^9^/L); 4) *D*-dimer level >4000 fibrinogen equivalent units (FEU); 5) positive anti-PF4 antibodies on Enzyme-linked immunosorbant assay (ELISA). Probable VITT was a case with *D*-dimer level >4000 FEU but one of the other criteria not met (timing, thrombosis, thrombocytopenia, or anti-PF4 antibodies) or, *D*-dimer level unknown or 2000–4000 FEU and all other criteria met. All cases of potential VITT after Ad26 were independently reviewed by the lead author of the UK case series paper to ensure comparability between the implementation of the case definition across both case series.

**Table 1:** Case definition criteria used to determine diagnostic certainty of potential cases of VITT

VITT	Description
Definite VITT	All five of the following criteria: • Onset of symptoms 5–30 days after vaccination against SARS-CoV-2 (or ≤42 days in patients with isolated deep vein thrombosis or pulmonary embolism) • Presence of thrombosis • Thrombocytopenia (platelet count <150 × 10^9^/L) • d-dimer level >4000 FEU • Positive anti-PF4 antibodies on ELISA
Probable VITT	d-dimer level >4000 FEU but one criterion not met (timing, thrombosis, thrombocytopenia, or anti-PF4 antibodies) or d-dimer level unknown or 2000–4000 FEU and all other criteria met
Possible VITT	d-dimer level unknown or 2000–4000 FEU with one other criterion not met, or two other criteria not met (timing, thrombosis, thrombocytopenia, or anti-PF4 antibodies)
Unlikely VITT	Platelet count <150 × 10^9^/L without thrombosis with d-dimer level <2000 FEU, or thrombosis with platelet count >150 × 10^9^/L and d-dimer level <2000 FEU, regardless of anti-PF4 antibody result, and an alternative diagnosis that is more likely.

ELISA, enzyme-linked immunosorbent assay; FEU fibrinogen-equivalent unit; PF4 platelet factor 4; VITT, vaccine-induced immune thrombocytopenia and thrombosis.

### ChAdOx1 case series

The UK case series was a prospective analysis that included 220 patients who presented to hospitals in the United Kingdom between March 22 and June 6, 2021 and who were classified as having definite or probable VITT using NICE case definition criteria (Table 1). All cases of VITT occurred after the first dose of ChAdOx1 vaccine^1^.

### Ethics oversight

The data set from Pavord et al., published in NEJM^1^, collected data via an anonymised electronic reporting form developed with Public Health England. This was completed for each patient by the local attending team. The data were anonymised, had no impact on patient care, and were reported as aggregate data.

For the case series of data from Johnson & Johnson, these data were from Janssen’s Global Safety Database, which captures data on adverse events as a pharmacovigilance requirement undertaken by drug and vaccine manufacturers globally, with no requirement for IRB approval. Data were anonymised prior to analysis, had no impact on patient care, and were reported as aggregate data.

### Statistics and reproducibility

A descriptive statistical analysis of definite or probable VITT cases (Table 1) was performed. Variables were described as numbers and percentages (based on non-missing data) or as medians and interquartile ranges (IQR). The variables studied were age, sex, race, country, the number of days since vaccination, comorbidities and risk factors for venous/arterial thrombosis, thrombocytopenia, and anti-PF4 antibody detection. Co-morbidities examined were autoimmune disease, previous thromboembolism, prothrombotic disorders (including thrombophilia and antiphospholipid syndrome), cancer, and concurrent SARS-CoV-2 infection. Relevant drug history that could influence coagulability, particularly the use of hormonal preparations and anticoagulants, were also assessed. Other variables were the type and location of thrombosis (including arterial thrombosis and thrombosis at multiple sites), the presence of intracranial haemorrhage, symptoms, outcome, and treatment modalities. Laboratory variables included platelet count, D-dimer level, the presence of anti-PF4 antibodies and standard coagulation parameters. An exploratory analysis compared categorical variables using Fisher’s exact test. Prognostic markers for fatal outcome were explored using a multivariate logistic regression model. No adjustment for multiplicity testing was implemented, which should be taken into consideration when interpreting the results.

Laboratory tests were performed in local laboratories. Normal ranges were determined at the local laboratories or with the thresholds used by the manufacturers of the reagents. Results for *D*-dimer levels are reported in FEUs. Positive thresholds for anti-PF4 antibodies were based on the manufacturers’ optical density thresholds or on locally derived normal ranges.

## Results

### Baseline and demographic features

When applying NICE criteria for VITT, we identified 86 cases of definite/probable VITT occurring in temporal association with Ad26 and 220 occurring after vaccination with ChAdOx1. The median age of patients with VITT after Ad26 was 43 years (IQR 34–52) and 68.6% were aged <50. The median age of patients with VITT after ChAdOx1 was 48 years (IQR 38–56) and 56% were aged <50 years (Table 2). The percentage of patients who were women was 55.3% after Ad26 and 55% after ChAdOx1. The geographical distribution of VITT cases after Ad26 is provided in Supplementary Table 1.

**Table 2:** Demographic and clinical features of patients with VITT after COVID-19 vaccination according to vaccine type

Characteristic	Ad26.COV2.S	ChAdOx1 nCoV-19	p-value^g^
	*N* = 86 n/N (%)^a^	*N* = 220 n/N (%)^a^	
Age Median (IQR)	43 (34–52)	48 (38–56)	<0.05
Age <50 years	59/86 (69)	122/218 (56)	NS
Age <60 years	79/86 (92)	185/218 (85)	NS
Sex			
Female	47 /85 (55)	119/217 (55)	NS
Risk factors			
for venous thrombosis^b^	37^c^/66 (56)	33/165 (20)	<0.0001
for arterial thrombosis^d^	39/66 (59)	31/165 (19)	<0.0001
for thrombocytopenia^e^	9/66 (14)	20/165 (12)	NS
for positive anti-PF4 antibody test	0/66	−	NT
SARS-CoV-2 PCR			
Positive	1/40 (3)	0/165	NS
Relevant drug history			
Heparin	0/60	0/165	NT
Antiplatelets	1/60 (2)	5/165 (3)	NS
Anticoagulants	0/60	7/165 (4)	NS
Hormonal therapy	6/61 (10)	11/165 (7)	NS
Steroids	1/60 (2)	−	NT
Other	0/60	−	NT
Pre-existing autoimmune disease	4/66 (6)	14/165 (8)	NS
Hashimoto’s thyroiditis/ hypothyroidism	2	4	
Autoimmune hepatitis	0	2	
Connective tissues disease	0	2	
Autoimmune hepatitis	0	2	
Crohn’s disease	0	2	
Vasculitis	1	0	
Multiple sclerosis	1	0	
Immune thrombocytopenic purpura	0	1	
Sarcoidosis	0	1	
Myasthenia gravis	0	1	
Guillain-Barré syndrome	0	1	

*CI* confidence interval, *IQR* interquartile range, *n/N* number of patients/number of patients with non-missing data, *NS* not significant (*p* > 0.05); *NT* not tested, *PCR* polymerase chain reaction.

^a^Percentage is calculated from the numbers of patients in each category for whom data were known.

^b^Active cancer, immobilisation, major surgery within 12 weeks, trauma, hormonal medications, obesity (body mass index >30 kg/m^2^), pregnancy, inherited thrombophilia (Factor V Leiden, protein *C* or *S* deficiency, antiphospholipid syndrome, antithrombin deficiency, prothrombin gene mutation), systemic lupus erythematosus, smoking, previous thromboembolic event, and concurrent COVID-19.

^c^Note that 1 patient tested positive for factor V Leiden and factor II G20210A mutations.

^d^Diabetes mellitus, hypertension, hyperlipidemia, obesity (body mass index >30 kg/m^2^), and smoking.

^e^Alcohol use disorder, autoimmune disease, bone marrow diseases (including aplastic anaemia, and leukaemia, myelodysplastic syndromes), liver function impairment, cancer treatment, enlarged spleen, exposure to toxic chemical, and viral infection (hepatitis C, cytomegalovirus, Epstein-Barr virus, and human immunodeficiency virus).

^f^Long exposure to heparin therapy, use of unfractionated heparin, orthopaedic surgery, cardiopulmonary surgery, and chronic bacterial infection such as periodontitis.

^g^Fisher’s exact test.

**Fig. 1:**
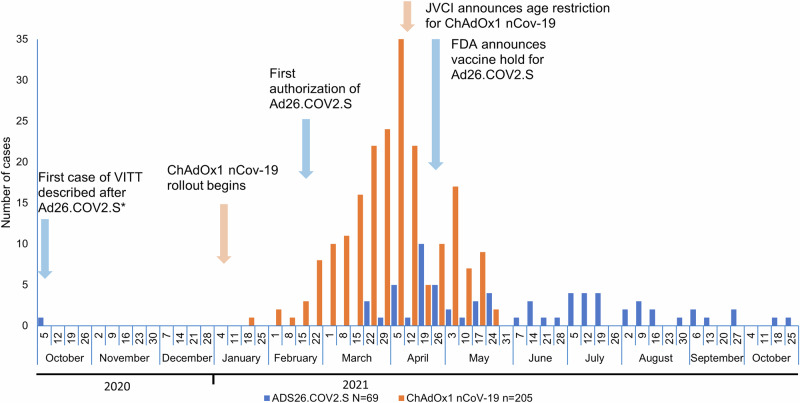
Sequence of events and distribution of VITT cases after Ad26.COV2.S (*n* = 69) and ChAdOx1 nCoV-19 (*n* = 205). Blue arrows refer to activities around Ad26.COV2.S, oange arrows indicate activities around ChAdOx1 nCoV-19. Columns show the number of VITT cases reproted over time for each vaccine. FDA, US Food and Drug Administration; JVCI, United Kingdom Joint Committee on Vaccination and Immunisation; VITT, vaccine-induced immune thrombocytopenia and thrombosis. *Reported during a clinical trial of Ad26.COV2.S

Cases of VITT after ChAdOx1 were reported from January 2021, peaked in March and April and were no longer collected after June 2021 (data cutoff date). Cases of VITT after Ad26 were dispersed between March and October 2021 (Fig. 1).

The percentage of patients with VITT after Ad26 who had risk factors for venous thrombosis or risk factors for other potential causes of thrombocytopenia (listed in Table 2) was 56.1% and 13.6%, respectively (not reported for ChAdOx1). Risk factors for arterial thrombosis were present in 59.1% of patients with VITT after Ad26 and 19% with VITT after ChAdOx1 (*p* < 0.0001). Only one patient with VITT (Ad26) was positive for SARS-CoV-2 by polymerase chain reaction. None of the patients with VITT after either vaccine had been exposed to heparin prior to presentation; 4% after ChAdOx1 had been on oral anticoagulants. Antiplatelet agents were reported for 1.7% and 3% of patients after Ad26 and ChAdOx1, and use of hormonal preparations for 9.8% and 6.5% of patients, respectively. One patient (Ad26) was taking steroids (unspecified). Four (6%) patients with VITT after Ad26 and 14 (8%) patients with VITT after ChAdOx1 had pre-existing autoimmune diseases (Table 2).

### Time-to-onset, presenting symptoms and outcome

The median time from vaccination (all post-dose 1) until time to onset in patients with VITT was 11 days (IQR 9–14) after Ad26 and 14 days (IQR 10–16) after ChAdOx1 (Table 3). The most commonly reported presenting symptoms in patients with VITT after Ad26 were headache (60%), nausea/vomiting (38.7%), fever (27.9%), abdominal pain (27.9%), body aches (24.6%), extremity pain (23%), hemiparesis (19.7%), chills (18%), dyspnoea (14.8%), chest pain (13.1%), myalgia (13.1%), reduced consciousness (13.1%), seizures (12.9%), blurred vision (11.5%) and extremity swelling (11.5%) (Supplementary Table 2). Case fatality was 29.9% in patients with VITT after Ad26 and 22% after ChAdOx1, with 68.4% and 62.5% of the fatal cases, respectively, occurring in women (Table 3).

**Table 3:** Type, location and outcome of thrombosis in patients with VITT after COVID-19 vaccination according to vaccine type

Characteristic	Ad26.COV2.S	ChAdOx1 nCoV-19^1^	*P*-value^d^
	*N* = 86	*N* = 220	
	n/N (%)^a^	n/N (%)^a^	
Days since vaccination			
Median (IQR)	11 (9–14)^b^	14 (10–16)^b^	NT
Outcome			
Not recovered	25/67 (37)	−	NT
Recovering	15/67 (22)	−	NT
Recovered	7/67 (10)	−	NT
Fatal	20/67 (30)	49/219 (22)	NS
Type of thrombosis			
Venous	71/86 (83)	173/220 (79)	NS
Arterial	2/86 (2)	−	NT
Mixed type	13/86 (15)	−	NT
Location of thrombosis			
Cerebral vein	43/85 (51) (all CVST)	110/220 (50) (all CVST)	NS
Splanchnic vein	22/82 (27) (including PVT)	41/220 (19) (including PVT)	NS
PE or DVT	46/82 (56)	82/220 (37)	<0.01
PE	32/82 (39)	63/220 (29)	NS
DVT	30/82 (37)	40/220 (18)	<0.01
Jugular vein	9/82 (11)	−	NT
Intracerebral artery	9/82 (11)	17/220 (8)	NS
Aortoiliac or extremity artery	6/82 (7)	26/220 (12)	NS
Splanchnic artery	3/82 (4)	−	NT
DIC	5/85 (6)	−	NT
Other	11/82 (13)	−	NT
Thrombosis at >1 anatomical region^c^	42/83 (51)	64/220 (29)	<0.001
CVT with extra-cerebral thrombosis^c^	23/43 (53)	−	NT
Secondary intracranial haemorrhage^c^	20/83 (24)	47/220 (21)	NS

*CVST* cerebral venous sinus thrombosis, *CVT* cerebral venous thrombosis, *DIC* disseminated intravascular coagulation, *DVT* deep vein thrombosis, *n/N* number of subjects/number of subjects with non-missing data, *NS* not significant (*p* > 0.05), *NT* not tested, *PE* pulmonary embolism, *PVT* portal vein thrombosis.

^a^Percentage is calculated from the numbers of patients in each category for whom data were known.

^b^All cases were after first dose of vaccine.

^c^DVT and PE were considered one location, internal jugular thrombosis and CVST were considered one location.

^d^Fisher’s exact test.

### Type and location of thrombosis

A majority of patients with VITT after Ad26 and ChAdOx1 developed venous thrombosis (82.6% and 79%, respectively), most frequently involving the cerebral veins and/or the dural venous sinuses (cerebral venous sinus thrombosis (CVST)). CVST was present in 50.6% of patients with VITT after Ad26 (74.4% were female) and 50% of patients with VITT after ChAdOx1 (60% were female). Deep-vein thrombosis and/or pulmonary embolism was present in 56.1% of patients after Ad26 and 37% after ChAdOx1 (*p* < 0.01). Splanchnic-vein thrombosis was reported for 26.8% of patients with VITT after Ad26 and 19% of patients with VITT after ChAdOx1. Aortoiliac thrombosis or extremity artery thrombosis occurred in 7.3% and 12% of patients, and intracerebral artery thrombosis occurred in 11% and 8% of patients, respectively (Table 3). Thrombosis was identified in multiple anatomical sites in 50.6% of patients after Ad26 and 29% of patients after ChAdOx1 (*p* < 0.001). Secondary intracranial haemorrhage occurred in 24.1% (75% female) and 21% (71% female) of patients, respectively. Secondary intracranial haemorrhage occurred in 18/43 (43%) patients with CVST after Ad26, and 40/110 (36%) patients with CVST after ChAdOx1.

### Laboratory findings

The median nadir platelet count was 41 × 10^9^/L (IQR 17–66 × 10^9^/L) in patients with VITT after Ad26 and 47 × 10^9^/L (IQR 28–76 × 10^9^/L) in patients with VITT after ChAdOx1. The median D-dimer level was 22,200 FEU and 24,000 FEU, and anti-PF4 antibodies were detected in 95.6% and 97%, respectively. Levels of fibrinogen, prothrombin time, and activated partial-thromboplastin time were within the normal ranges and were similar in each vaccine group (Table 4).

**Table 4:** Laboratory findings in patients with VITT after COVID-19 vaccination according to vaccine type

Characteristic	Ad26.COV2.S	ChAdOx1 nCoV-19^1^
	*N* = 86	*N* = 220
Platelet count (cells × 10^9^/L) median (IQR)	41.0 (17.0–66.0) nadir	47.0 (28.0–76.0) at admission
D-dimer level (ng/mL) median (IQR)	22,228 (9819–36,675)	24,000 (8000–37,000) at admission
Fibrinogen level (g/L) median (IQR)	1.46 (1.10–2.39) nadir	2.2 (1.2–3.1) at admission
Prothrombin time (sec) median (IQR)	15.0 (13.0–18.6)	13 (10–14) at admission
Activated partial-thromboplastin time (sec) median (IQR)	31.0 (26.0–34.0)	29 (22–30) at admission
Anti-PF4 antibodies (OD) median (IQR)	2.46 (1.986–2.702)	−
Positive anti-PF4 antibodies n/N (%)	65/68 (96%)	198/204 (97%)
Positive functional platelet test (not specified) n/N (%)	2/10 (20%)	−

IQR, interquartile range; OD, optical density; PF4, platelet factor 4, VITT, vaccine-induced immune thrombocytopenia and thrombosis.

The normal ranges for variables are as follows: platelets 150–400 × 10^9^/L, D-dimer <500 ng/mL FEU, fibrinogen 1.5–4.0 g/L, prothrombin time 10.0–12.0 s, activated partial-thromboplastin time 25.0–37.0 s, anti-PF4 antibodies <0.400 OD.

### Treatment modalities

Non-heparin-based anticoagulation was the mainstay of treatment; however heparin was administered at some point during admission in 35.8% of patients with VITT after Ad26 and 23% after ChAdOx1. Intravenous immunoglobulin was given to 66.7% and 72% of patients, respectively, plasma exchange to 4.0% and 8.0%, platelet transfusion to 9.8% and 14% and thrombectomy to 32.7% and 15% of patients, respectively (Supplementary Table 3).

### Prognostic markers for fatal outcome

In patients with VITT after ChAdOx1, increasing risk of death was related to lower platelets, lower fibrinogen, higher D-dimer and presence of CVST. Independent risk factors for mortality were platelet count nadir <30 × 10^9^/L and the presence of intracranial haemorrhage^1^. For patients vaccinated with Ad26, country, secondary intracranial haemorrhage and low platelet count nadir were related to fatal outcome, with intracranial haemorrhage and platelet count nadir remaining significant in the multivariate analysis (Supplementary Table 4). No treatment modality in the Ad26 cohort correlated significantly with survival.

## Discussion

To our knowledge, this is the largest case series of VITT following Ad26 yet published and the first side-by-side investigation of the features of VITT after Ad26 and ChAdOx1. Both are adenovirus-vectored vaccines used extensively during the SARS-CoV-2 pandemic that saved many millions of lives from COVID-19^19^. Albeit very rare, VITT was a devastating complication of these two vaccines, and it is important to understand if there were differences between them in their clinical features. Strengths of this comparison include the prospective collection of data from reported cases in one case series, and adjudication of cases reported in the post-marketing setting by the same expert to ensure consistency of approach to disease classification. Weaknesses include potential for reporting bias of worse cases, reliance on retrospective data for VITT after Ad26, and the variation in the use of vaccine and available resources for diagnosis and treatment in different countries using the Ad26 vaccine. Hence, incidence of VITT after the two vaccines is not compared.

Given the very different methods by which the clinical information was acquired, the clinical features of VITT after the two vaccines are striking in their similarity. The few differences observed can be explained by the possible differences in recommendations for use, timing of awareness of VITT, and the speed and availability of resources for diagnosis and management. For example, thrombosis in more than one anatomical region was observed to be higher in patients with VITT following Ad26 than after ChAdOx1 (50.6 vs 29%), and likely reflects differences in thrombosis detection rates as a function of the type and extent of radiological imaging, which evolved as awareness of the potential for multisite thromboses increased. It is notable that, where additional imaging was carried out in asymptomatic sites for screening purposes, occult thrombosis was identified in 83% after ChAdOx1^20^. The observed lower fatality rate due to VITT after ChAdOx1 administration versus Ad26 and the differences in the use of treatment modalities may reflect the rapid clinical network established in the UK once this ChAdOx1 complication became apparent^8^. Prospective identification of these cases ensured rapid diagnosis, investigation, and treatment^1^. By contrast, cases following Ad26 were spontaneously reported to Janssen from multiple countries, including the United States, 12 countries in Europe, South Africa, and Brazil; each with different healthcare systems and variable patient access to facilities with the necessary testing and treatment resources. This is reflected by a significant correlation between outcome and country in the univariate analysis. Furthermore, fatal cases are more likely to be reported spontaneously than non-fatal or milder cases, potentially resulting in a reporting bias toward more fatal cases after Ad26. Prognostic markers for fatal outcome common to both vaccines were the platelet nadir and the presence of intracranial haemorrhage. Finally, patients with VITT after Ad26 were younger than those with VITT after ChAdOx1, however this is strongly influenced by the populations targeted for vaccination, which are likely to have differed across the countries where they were used, and which evolved over time. While this finding could indicate a difference between VITT after Ad26 versus ChAdOx1, it cannot be interpreted in the absence of data on the age of the vaccinated populations.

In this study, 19 of patients with VITT after Ad26 and 50 after ChAdOx1 received anticoagulation with heparin, and 5 and 30 patients respectively, received platelet transfusion. Early reports suggested poorer outcomes following use of heparin anticoagulation^21^. However, only around 5% of patients with VITT have cross-reacting anti-PF4/heparin antibodies and heparin anticoagulation is therefore likely to be safe in the majority of cases^22,23^. Platelet transfusion is usually avoided based on experience with HIT but is sometimes indicated after assessment of benefit versus risk in individual patients^5,22^. We observed no correlation between the use of heparin and a fatal outcome.

The overall frequency of autoimmune diseases lies within the range reported as lifetime prevalence in the general population^24–27^. However, a range of rare autoimmune diseases was observed in patients who later developed VITT after either vaccine, at a frequency that appears to be higher than expected given the sample size. Research is required to further explore this observation and the potential involvement of immune-regulatory genes.

A key strength of this analysis is the use of the same expert to classify the disease after both vaccines, reducing the possibility of misinterpretation. Potential limitations include the use of different data sources, specifically, post-marketing surveillance for Ad26 versus a prospective case series for ChAdOx1, which may have affected case detection rates, hence incidence is not compared. Thoroughness of the investigations undertaken at the height of the pandemic when healthcare systems were under severe strain may have varied, as did the approach to treatment. Both Ad26 and ChAdOx1 were administered in different countries according to local recommendations. As a result, differing age groups and risk groups were exposed to each vaccine and these practices may have modified the risk of VITT. Exposure data are limited and do not allow robust calculation of incidence rates. Underreporting of VITT cases early on after vaccine rollout may have occurred, whereas increasing awareness of the phenomenon of VITT over time may have caused reporting bias for VITT following Ad26 which was introduced after ChAdOx1. Finally, we did not compare continuous variables as those were reported using median and range and non-parametric rank tests for medians require access to subject-level data which we did not have for patients who received ChAdOx1. The data collection process was conducted under diverse conditions, and the presence of significant p-values does not necessarily indicate a significant difference in the presentation of the condition being tested. Similarly, a non-significant value should not be automatically interpreted as indicating an equal frequency between the variables under comparison. It is important to consider the potential differences in collection conditions, such as variations in clinical practice, timing, or the amount of information recorded, which could influence the observed values and frequencies.

## Conclusion

Within the limitations of the available data, our study finds no strong evidence for a clinically relevant difference in VITT occurring after Ad26 or ChAdOx1. We consider that the observed differences between vaccines in age, risk factors and patterns of thrombosis are likely to result from the demographic of the populations vaccinated, and the situational and reporting differences in how, when and where patients were identified and treated. Data from this large case series contributes to our understanding of VITT, and research is ongoing to identify the genetic risk factors and pathological processes. Vaccination against SARS-CoV.2 continues to be the most effective way to reduce or prevent severe or fatal disease.

## Supplementary information

Supplementary information

Description of additional supplementary files

Supplementary data 1

## Data Availability

All data pertaining to cases of VITT occurring after ChAdOx1 nCoV-19 were obtained from published data. Data from Johnson & Johnson’ Global Safety Database are not publicly available for sharing. The source data for Fig. 1 is in Supplementary Data 1. Requests for sharing can be sent to the Corresponding Author and will be evaluated on an individual basis.
