# Supplementary material for: Comparison of vaccine-induced immune thrombocytopenia and thrombosis cases following two adenovirus-vectored COVID-19 vaccines
Source: Commun Med (Lond). 2025 May 10;5:168. doi: 10.1038/s43856-025-00891-x (PMC12065847; doi:10.1038/s43856-025-00891-x)
Supplement: Supplementary file 1 — Supplementary information [file 43856_2025_891_MOESM1_ESM.pdf]

Comparison of vaccine-induced immune thrombocytopenia and thrombosis (VITT) cases following two adenovirus-vectored COVID-19 vaccines

Rian Van Rampelbergh, Sue Pavord, Luis Anaya-Velarde, Vitalija van Paassen, Karin Hardt, Emiliano Tatar, Javier Ruiz-Guiñazú, Dawn Baumgardner, Valérie Oriol Mathieu, Nicolas Praet, Hendy Kristyanto, Jerald Sadoff, Macaya Douoguih, Yimei Xu, Frank Struyf

SUPPLEMENT

List of tables

Supplementary Table 1 Geographic distribution of patients with VITT after Ad26.COV2.S vaccination 2

Supplementary Table 2 Presenting symptoms in patients with VITT after Ad26.COV2.S vaccination 3

Supplementary Table 3 Treatments used in patients with VITT after COVID-19 vaccination according to vaccine type 4

Supplementary Table 4 Demographics, clinical characteristics, and laboratory findings in patients with definite/probable VITT after Ad26.COV2.S significantly related to outcome 5

Supplementary Table 1 Geographic distribution of patients with VITT after Ad26.COV2.S vaccination

**Characteristic**	**Ad26.COV2.S N=86 n (%)**
Austria or Germany	6 (7.0)
Belgium	1 (1.2)
Brazil	2 (2.3)
Denmark	1 (1.2)
France	2 (2.3)
Greece	1 (1.2)
Italy	5 (5.8)
Poland	1 (1.2)
Portugal	2 (2.3)
Slovenia	2 (2.3)
South Africa	1 (1.2)
Spain	5 (5.8)
The Netherlands	3 (3.5)
US	49 (57.0)
Unknown (EU)	5 (5.8)
Total EU	34 (39.5)

VITT, vaccine-induced immune thrombocytopenia and thrombosis

Supplementary Table 2 Presenting symptoms in patients with VITT after Ad26.COV2.S vaccination

Characteristic	Ad26.COV.2.S N=86 n/N (%)*
**General**	
Fever	17 / 61 (27.9)
Chills	11 / 61 (18.0)
Body aches	15 / 61 (24.6)
Myalgia	8 / 61 (13.1)
**Neurologic**	
Headache	42 / 70 (60.0)
Hemiparesis	12 / 61 (19.7)
Blurred vision	7 / 61 (11.5)
Diplopia	3 / 61 (4.9)
Seizures	8 / 62 (12.9)
Reduced consciousness	8 / 61 (13.1)
Aphasia	2 / 61 (3.3)
**Gastrointestinal**	
Nausea/Vomiting	24 / 62 (38.7)
Abdominal pain	17 / 61 (27.9)
**Respiratory**	
Chest pain	8 / 61 (13.1)
Dyspnoea	9 / 61 (14.8)
Haemoptysis	2 / 61 (3.3)
Cough	1 / 61 (1.6)
**Extremities**	
Extremity pain	14 / 61 (23.0)
Leg swelling	7 / 61 (11.5)
**Bleeding tendency**	
Bruising	5 / 61 (8.2)
Other	0 / 61

n/N, number of patients / number of patients with non-missing data
*Percentage is calculated from the numbers of patients in each category for whom data were known.

Supplementary Table 3 Treatments used in patients with VITT after COVID-19 vaccination according to vaccine type

**Characteristic**	**Ad26.COV.2.S N=86 n/N (%)***	**ChAdOx1 nCoV-19[1] N=220 n/N (%)***
Non-heparin anticoagulant	43 / 52 (82.7)	150 / 220 (68)
Heparin	19 / 53 (35.8)	50 / 220 (23)
Intravenous Immunoglobulin	36 / 54 (66.7)	158 / 220 (72)
Corticosteroids	21 / 52 (40.4)	58 / 220 (26)
Platelet transfusion	5 / 51 (9.8)	30 / 220 (14)
Plasmapheresis	2 / 50 (4.0)	17 / 220 (8)
Thrombus removal (thrombectomy, thrombolysis, etc.)	17 / 52 (32.7)	32 / 220 (15)
Other treatment	0 / 50	-

n/N, number of patients / number of patients with non-missing data
*Percentage is calculated from the numbers of patients in each category for whom data were known.

Supplementary Table 4 Demographics, clinical characteristics, and laboratory findings in patients with definite/probable VITT after Ad26.COV2.S significantly related to outcome

	Fatal N=20	Outcome Non-fatal N=47	Unknown N=19	p-value*
**Country**				
N	20	47	19	<0.0001
Austria	1 (5.0%)	1 (2.1%)	0	
Belgium	1 (5.0%)	0	0	
Brazil	2 (10.0%)	0	0	
Denmark	0	0	1 (5.3%)	
France	0	1 (2.1%)	1 (5.3%)	
Germany	3 (15.0%)	1 (2.1%)	0	
Greece	0	1 (2.1%)	0	
Italy	1 (5.0%)	1 (2.1%)	3 (15.8%)	
Poland	1 (5.0%)	0	0	
Portugal	2 (10.0%)	0	0	
Slovenia	1 (5.0%)	1 (2.1%)	0	
South Africa	0	1 (2.1%)	0	
Spain	3 (15.0%)	2 (4.3%)	0	
The Netherlands	0	3 (6.4%)	0	
USA	4 (20.0%)	33 (70.2%)	12 (63.2%)	
Unknown (EU)	1 (5.0%)	2 (4.3%)	2 (10.5%)	
**Secondary intracranial haemorrhage-no./total no. (%)**				
N	19	47	17	<0.05
Yes	10 (52.6%)	9 (19.1%)	1 (5.9%)	
No	9 (47.4%)	38 (80.9%)	16 (94.1%)	
Unknown	1	0	2	
**Platelet count, nadir ($\times 10^9$/L)**				
N	20	47	19	<0.05
Mean	34.5	59.1	46.9	
95% CI	19.2; 49.7	46.7; 71.4	30.1; 63.8	
Median	27.0	58.0	32.0	
Range	(7.0; 135.0)	(6.0; 140.0)	(7.0; 135.0)	
IQ range	(12.5; 44.0)	(20.0; 91.0)	(18.0; 66.0)	

Percentage is calculated from the numbers of patients in each category for whom data were known.
* p-values using t-test for continuous outcomes, Fisher exact test for categorical outcomes. Only known outcomes are included in testing

References

1. Pavord, S., *et al.* Clinical features of vaccine-induced immune thrombocytopenia and thrombosis. *N Engl J Med* **385**, 1680-1689 (2021).
