# Supplementary material for: Comparison of vaccine-induced immune thrombocytopenia and thrombosis cases following two adenovirus-vectored COVID-19 vaccines
Source: Commun Med (Lond). 2025 May 10;5:168. doi: 10.1038/s43856-025-00891-x (PMC12065847; doi:10.1038/s43856-025-00891-x)
Supplement: Supplementary file 2 — Description of additional supplementary files [file 43856_2025_891_MOESM2_ESM.pdf]

**Description of Additional Supplementary Files**

File name- Supplementary Data 1

File description- The source data for Figure 1 is in Supplementary Data 1
